# Ohio State Board of Examiners, 1897

**Published:** 1897-09

**Authors:** 


### EXAMINATION QUESTIONS.

### OHIO STATE BOARD OF EXAMINERS, 1897.

1. Name the muscles surrounding the femur of a horse, give their point of attachment and their point of insertion, and state their action; also state what muscles are inserted to the superior and inferior trochanters. 2. Describe the pneumogastric nerve (nervus vagus). 3. Name and describe the metatarsal bones of an ox (from tarsus to toe). 4. Describe the stomach of a horse and give its position when full of food. 5. Describe the process of digestion in a ruminating animal; follow the food from mouth to anus, and state the changes it undergoes. 6. What forces are acting in effecting inspiration and expiration? 7. Describe the characteristic histological structure of the kidneys. 8. Give the natural history, as far as known, of sclerostomum equinum (strongylus armatus); state how, and in which way, it produces injury to its host and may become the cause of disease. 9. State the source, and explain the cause, of the development of abnormal degrees of temperature in feverish diseases. 10. Name, and briefly describe, three poisonous umbelliferæ growing wild in the United States. 11. What is "heaves?" Give a definition. 12. Describe the essential symptoms and also the characteristic morbid changes of (contagious) pleuro-pneumonia of cattle, particularly state the differences between this disease and bovine tuberculosis. 13. Give a brief description of distemper (also called strangles) of horses; give all the characteristics, state the cause or causes, and briefly delineate a rational treatment. 14. If in a slaughtered animal, a steer for instance, you should find that in the bones, particularly in the femur and humerus, the narrow and the more porous parts abnormally red, and the marrow spaces and canals enlarged, what would be your diagnosis? Would you condemn the meat or not? 15. Give the characteristic symptoms of spavin, and state the means by which a diagnosis can be secured. 16. Give the various methods of treatment applied to an umbilical hernia; state your preference and give reasons therefor. 17. How would you treat an inveterate or neglected case of fistulous withers? 18. State the effect of tartar emetic upon our various domestic animals. 19. Describe the symptoms of arsenic-poisoning, and give the treatment you would apply if called in time. 20. State the pharmacognostic properties of aloes, the essential difference between the principal kinds in the market, and their effects upon our domestic animals, particularly horses. 21. What is a bacterium? What a bacillus? What a micrococcus? What is meant by a pathogenic bacterium, and what by a saprophytic bacterium? What by an aërobic, and what by an anaërobic bacterium? 22. What different means may be employed to secure the diagnosis of glanders, and which is the surest and most reliable? 23. How would you proceed in a case of obstetrics if there are twin calves, one with a posterior and the other with an anterior presentation, and both equally far advanced? 24. If a cow has not cleaned in due time after calving, what would you do and how would you proceed to remove the afterbirth? 25. How would you shoe a horse with pumiced feet and convex soles? 26. Suppose you should be called upon to select a saddle-horse for a wealthy gentleman weighing 175 pounds, a good horseman, and desirous of getting a horse that possesses speed and endurance, what kind of a horse would you select? State particulars (so-called points) you would demand. 27. At what age appears the roundish form ($4\frac{1}{2}$ to $4\frac{1}{2}$) in the friction-surface of the middle teeth of the lower jaw of the horse, provided the incisors are of normal length? 28. Suppose a hog is butchered and is found to be trichinous, all the trichinæ are encysted and a deposit of lime-salts has taken place in some of the cysts, while others are not only free from lime-salts but also from fat-cells at their poles, how would you account for the difference, and how long must it be since the animal became infected? 29. Describe the characteristics of a thoroughbred horse. 30. Describe the characteristics of a short-horned cow.

---

Veterinarian Knowles, of Montana, fully alive to the various duties of his position, has been investigating the cause of death of a number of cattle and sheep, and finds larkspur or wolfsbane, a species of aconite, sometimes called monkshood, botanical name delphinium. In a circular letter bearing upon the subject he forcibly reminds the interests involved that owners of live-stock and organizations connected therewith must lend a helping and encouraging hand in this work for the best results.

